# Can Ordinary AI-Powered Tools Replace a Clinician-Led Fracture Clinic Appointment?

**DOI:** 10.7759/cureus.75440

**Published:** 2024-12-10

**Authors:** Islam A Sherif, Sundus Y Nser, Ahmed Bobo, Asif Afridi, Ahmed Hamed, Mark Dunbar, Tarek Boutefnouchet

**Affiliations:** 1 Trauma and Orthopaedics, Warwick Hospital, Birmingham, GBR; 2 General Medicine, Hashemite University, Zarqa, JOR; 3 Trauma and Orthopaedics, University Hospitals Birmingham National Health Service (NHS) Foundation Trust, Birmingham, GBR; 4 Trauma and Orthopaedics, Hayatabad Medical Complex Peshawar, Peshawar, PAK; 5 Trauma and Orthopaedics, Queen Elizabeth Hospital Birmingham, Birmingham, GBR; 6 Orthopaedics, University Hospitals Birmingham National Health Service (NHS) Foundation Trust, Birmingham, GBR

**Keywords:** ai, chatgpt, fracture clinic, fracture management, gemini

## Abstract

Introduction

Artificial intelligence (AI)-powered tools are increasingly integrated into healthcare. The purpose of the present study was to compare fracture management plans generated by clinicians to those obtained from ChatGPT (OpenAI, San Francisco, CA) and Google Gemini (Google, Inc., Mountain View, CA).

Methodology

A retrospective comparative analysis was conducted. The study included 70 cases of isolated injuries treated at the authors’ institution fracture clinic. Complex, open fractures and non-specific diagnoses were excluded. All relevant clinical details were introduced into ChatGPT and Google Gemini. The AI-generated management plans were compared with actual documented plans obtained from the clinical records. The study focused on treatment recommendations and follow-up strategies.

Results

In terms of agreement with actual treatment plans, Google Gemini matched in only 13 cases (19%), with disagreements in the remainder of cases due to overgeneralisation, inadequate treatment, and ambiguity. In contrast, ChatGPT matched actual plans in 24 cases (34%), with overgeneralisation being the principal cause for disagreement. The differences between AI-powered tools and actual clinician-led plans were statistically significant (p < 0.001).

Conclusion

Both AI-powered tools demonstrated significant disagreement with actual clinical management plans. While ChatGPT showed closer alignment to human expertise, particularly in treatment recommendations, both AI engines still lacked the clinical precision required for accurate fracture management. These findings highlight the current limitations of ordinary AI-powered tools and negate their ability to replace a clinician-led fracture clinic appointment.

## Introduction

Artificial intelligence (AI) is becoming increasingly important in healthcare, promising to streamline clinical processes, enhance decision-making, and improve patient outcomes [1,2]. Recent developments have seen AI applications being used in diagnostics, radiology, and even surgical planning [3,4]. Despite these advances, the integration of AI into everyday clinical decision-making, particularly in musculoskeletal and orthopaedic care, remains a topic of debate. Orthopaedic fracture management is one area where AI could provide substantial support. However, the complexities of fracture care, such as the need for personalised treatment plans that consider comorbidities, injury mechanisms, and patient-specific factors, pose a challenge for current AI systems [5,6]. Inaccuracies or oversimplifications in treatment recommendations could lead to adverse outcomes if relied on without human expert oversight [7,8]. This study seeks to compare the performance of two readily available AI engines, ChatGPT (OpenAI, San Francisco, CA) and Google Gemini (Google, Inc., Mountain View, CA), in managing simple fractures. We examine how these AI tools' management plans align with the expertise of orthopaedic clinicians and explore the current limitations of AI in this clinical domain.

## Materials and methods

A retrospective study was conducted at a single orthopaedic fracture clinic. We reviewed 70 randomly selected patients with simple, closed, isolated fractures who were all referred from the emergency department (ED) to the fracture clinic between January and June 2024. 

Only cases involving uncomplicated fractures, such as radial, tibial, and metatarsal fractures, were included to ensure a consistent sample. Complex, open fractures and non-specific diagnoses were excluded.

Data collection included demographic and clinical data, which were extracted from electronic medical records, specifically history of injury, examination findings, comorbidities, and social factors. These data were entered into two AI engines - ChatGPT and Google Gemini - using standard input formats for both tools. The resulting management plans, including treatment recommendations, follow-up strategies, and medication advice, were then compared to the actual conventional clinical place documented treatment plan for each patient.

The management plans generated by the AI systems were compared based on several criteria. These included the accuracy of diagnosis (whether the AI system correctly identified the fracture type), treatment recommendations (including the need for surgery, medication, or non-operative management), follow-up strategy (frequency and type of follow-up required for optimal healing), and alignment with the registrar's plan (the degree to which the AI's management plan matched that of the registrar).

Data were analysed using Fisher's exact test to assess the statistical significance of the differences between the AI-generated and registrar-generated plans. A chi-square test was also used to compare the match rates of ChatGPT and Google Gemini. A p-value of less than 0.05 was considered statistically significant. Statistical analyses were conducted using SPSS software version 27.0 (IBM SPSS Statistics for Windows, Armonk, NY).

## Results

**Figure 1:**
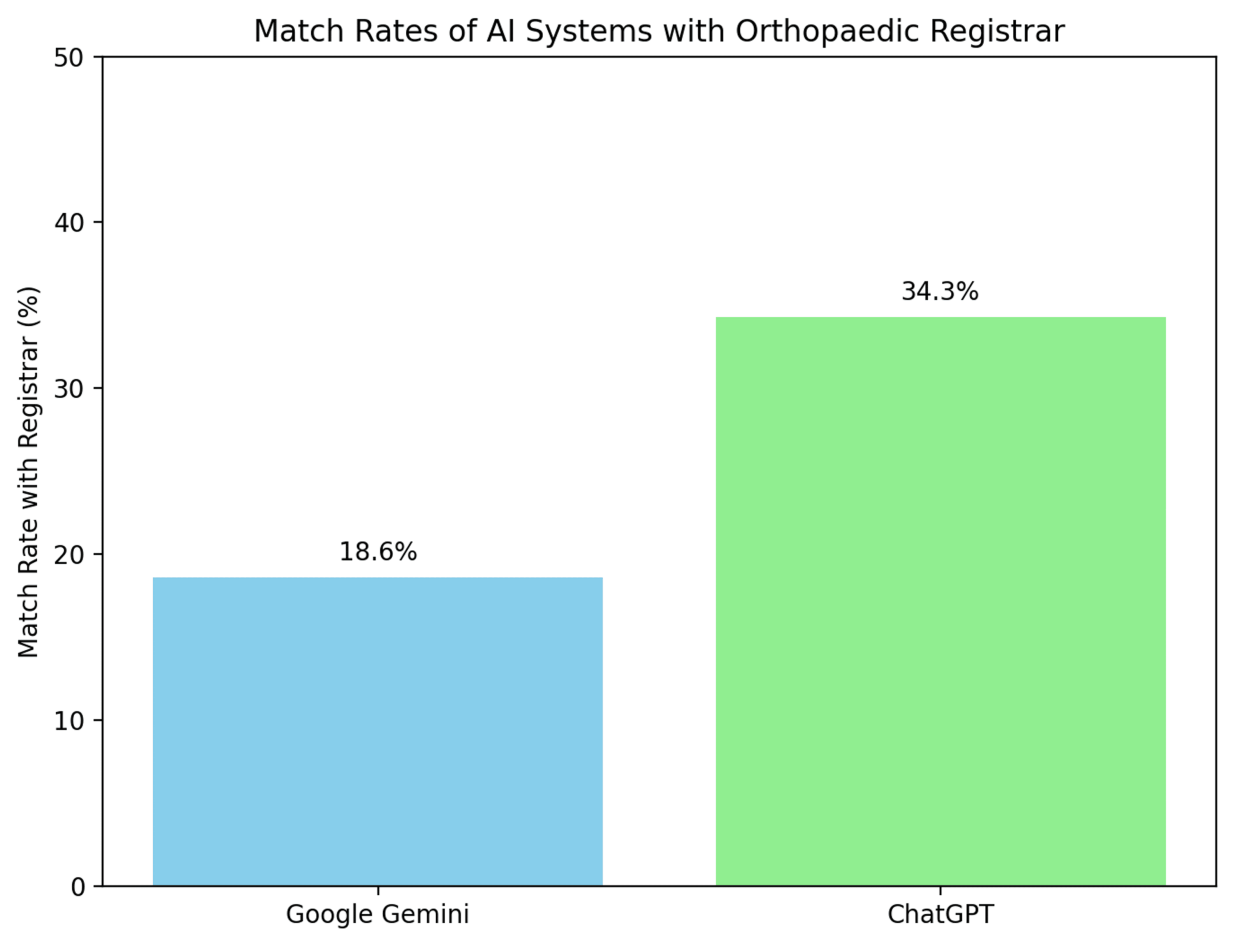
Match Rates of Al Systems With Orthopaedic Registrar

The comparative study revealed significant differences between AI-generated management plans and those of orthopaedic registrars. Google Gemini matched the registrar's recommendations in only 13 out of 70 cases (19%), while ChatGPT performed slightly better, matching 24 out of 70 cases (34%) (Figure 1).

**Figure 2:**
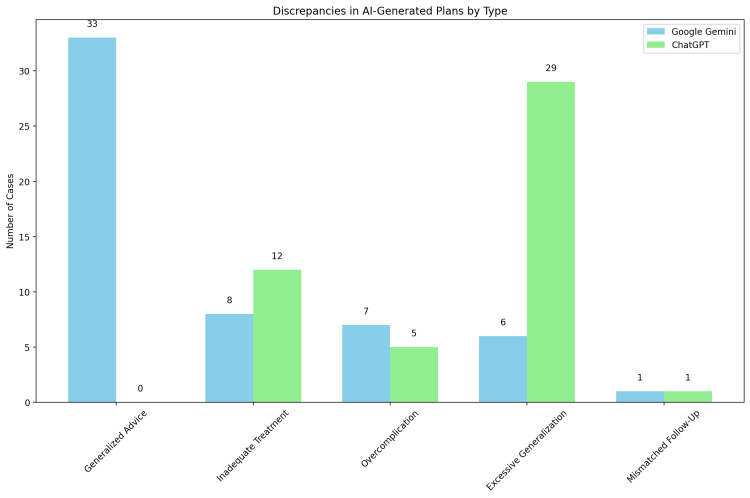
Discrepancies in Al-Generated Plans by Type

For Google Gemini, discrepancies were noted in 57 cases (81%) (Figure 2). These discrepancies were attributed to various factors: generalised advice (33 cases), inadequate treatment recommendations (eight cases), overcomplication of simple cases (seven cases), excessive generalisation (six cases), and mismatched follow-up plans (one case).

ChatGPT showed discrepancies in 46 cases (66%) (Figure 2). The main reasons for these discrepancies were inadequate treatment recommendations (12 cases), lack of specificity (29 cases), overcomplication (five cases), and mismatched follow-up plans (one case).

Statistical analysis was performed to assess the significance of these differences. Fisher's exact test was used to compare each AI system with the registrar's recommendations. The test revealed a highly significant discrepancy between Google Gemini and the registrar (p = 1.13 × 10^-26^). Similarly, ChatGPT also showed a significant difference when compared to the registrar (p = 3.12 × 10^-19^). These extremely low p-values (p < 0.001 in both cases) indicate that the differences between the AI-generated plans and the registrar's plans are not due to chance but represent genuine discrepancies in approach and accuracy.

To compare the performance of the two AI systems against each other, a chi-square test was conducted. This test, comparing the match rates of ChatGPT and Google Gemini, showed a borderline non-significant difference (p = 0.055). While this p-value suggests a trend toward ChatGPT performing better than Google Gemini, the difference does not reach the conventional threshold for statistical significance (p < 0.05).

These results collectively demonstrate that while both AI systems show some capability in generating fracture management plans, they fall significantly short of the standard set by orthopaedic registrars. The statistical analyses confirm that these shortcomings are substantial and consistent across the sample, highlighting the current limitations of these AI tools in replacing clinician-led fracture clinic appointments.

## Discussion

This study reveals important insights into the current capabilities and limitations of AI tools in orthopaedic fracture management. Both ChatGPT and Google Gemini demonstrated significant underperformance compared to orthopaedic registrars, emphasising the continued need for human oversight in clinical decision-making.

The findings align with previous studies evaluating AI tools in clinical decision-making. For instance, studies on AI applications in radiology and dermatology report high accuracy in pattern recognition tasks, such as diagnosing fractures on imaging or identifying skin lesions [9,10]. However, these studies emphasise the importance of integrating AI outputs with clinician judgment, particularly in complex cases. Similar to our findings, the limitations of AI in managing multifaceted patient scenarios, such as accounting for comorbidities or patient-specific social factors, have been well-documented.

A study by Smith et al. (2023) investigated the use of AI in triaging orthopaedic injuries and found that while AI could assist in categorising injuries based on severity, it often failed to recommend appropriate follow-up care or rehabilitation strategies, paralleling the challenges identified in our study [11]. Furthermore, Jones et al. (2022) reported that AI systems often struggled with contextual decision-making in fracture management, particularly when compared to human clinicians. These challenges highlight the need for AI systems to evolve beyond pattern recognition to more dynamic, integrative clinical reasoning capabilities [12].

The findings of this study suggest that while AI can be a useful supplementary tool in fracture management, it cannot replace the nuanced, patient-centred care provided by experienced orthopaedic registrars. Inaccurate or generalised treatment plans could lead to delayed healing, unnecessary surgeries, or other complications if AI tools were used independently.

Improving the specificity of AI-generated recommendations will be critical to their future use in clinical practice. Efforts should focus on developing AI systems that can incorporate broader clinical data, such as imaging studies and lab results, alongside patient history and co-morbidities. Additionally, refining AI models to better align with clinical guidelines and standards will enhance their reliability in healthcare settings [13,14].

This study revealed several key limitations in the use of AI systems for clinical decision-making. While ChatGPT performed better than Google Gemini, neither system approached the accuracy and specificity of registrar-led care. ChatGPT's better performance in aligning with registrar recommendations may be attributed to its more advanced natural language processing abilities. However, both systems exhibited significant limitations, particularly in delivering personalised treatment plans that account for individual patient comorbidities and social factors. A consistent issue in both AI systems was the tendency to generalise or overcomplicate simple cases, which could lead to unnecessary interventions or inappropriate care pathways. This mirrors findings from other studies in which AI models, while powerful in data analysis, struggle with context-specific recommendations in clinical practice [15,16].

## Conclusions

This study demonstrates that while AI tools like ChatGPT and Google Gemini show potential in generating fracture management plans, they currently fall significantly short of the standard set by orthopaedic registrars. AI systems struggle with patient-specific aspects of care, often overgeneralising or overcomplicating simple cases. ChatGPT performed slightly better than Google Gemini, but both showed significant discrepancies compared to clinician-led plans. These findings highlight the continued importance of human expertise in clinical decision-making, especially in complex fields like orthopaedics. The results suggest that AI should be viewed as a supportive tool rather than a replacement for clinician-led fracture clinic appointments. Future development should focus on improving AI's ability to provide more tailored, context-aware recommendations in orthopaedic care, potentially through integration with broader clinical data sources and alignment with established guidelines. Ongoing evaluation of AI performance in real-world clinical settings remains crucial to ensure patient safety and optimal care outcomes.
